# A social cognitive perspective on occupational identity development in college students

**DOI:** 10.1186/s41155-022-00215-1

**Published:** 2022-10-25

**Authors:** Jaisso Vautero, Ana Daniela Silva

**Affiliations:** 1 Pro-Rectory of Student Affairs, Federal University of Santa Maria, Av. Roraima, 1000, Santa Maria, RS 97105-900 Brazil; 2 School of Psychology, University of Minho, Braga, Portugal

**Keywords:** Occupational identity, College students, Academic satisfaction, Academic self-efficacy, Goal progress

## Abstract

Occupational identity is a central concept of career development, by providing a sense of direction and meaning across career development. This study aimed to examine how this concept can be associated with career processes through model formed by a set of socio-cognitive factors. The participants were 358 college students at a Brazilian university who completed measures of occupational identity, environmental supports and barriers, self-efficacy, goal progress, and academic satisfaction. Analysis indicates that the occupational identity status was partially well predicted by the combination of self-efficacy to cope with barriers, supports, academic satisfaction, and goal progress. These results highlight that students with a positive sense of competencies to deal with barriers and adequate levels of academic satisfaction would easily establish an occupational identity.

Occupational identity provides a sense of direction and meaning and helps to establish a framework to define occupational goals (Skorikov & Vondracek, 1998). Erikson (1968, 1998) posited that the main psychosocial task in transition from adolescence to adulthood is the identity crises resolution, meaning reaching an identity sense. The successful resolution of this crisis depends on the ego’s capability to synthesize significant identifications, futures aspirations, and opportunities available in social roles (Vautero et al., 2018). This process of identity formation is based on the examination of alternatives and commitments to meaningful choices among such ego identity elements as occupation (Dellas & Jernigan, 1981). Occupational merges as a central concept of career development (Skorikov & Vondracek, 1998); however, in recent years, few researches have been conducted to examine this process. Social cognitive process, on the other hand, merges as a central aspect in career development (Sheu & Bordon, 2017), helping to explain how people make choices, develop, perform, and gather satisfaction in the career domain (Lent, 2013). Previous works have associated occupational identity with a number of socio-cognitive constructs, like self-efficacy (e.g., Choi et al., 2012; Germeijs & Verschueren, 2009), academic progress or outcomes (Guan et al., 2015; Hirschi, 2013; Steiner et al., 2019), academic satisfaction (e.g., Byars-Winston & Rogers, 2019; Steiner et al., 2019), and self-efficacy and wellbeing (Green, 2020). These associations among sociocognitive constructs and occupational identity points to the possibility to conceive a hypothetical structure to predict occupational identity achievement based on and environmental (i.e., supports and barriers) and influence social cognitions (i.e., self-efficacy and goal progress perception). In this sense, this study aimed to examine previous evidence and propose a model that assumes occupational identity as an indicator of career development. Such model posits the occupational identity can be represented as result of the interactions among a set of socio-cognitive constructs.

## Occupational identity

The process of identity development is based on the examination of alternatives and commitments with meaningful choices in many identity domains, such as occupational, political and relational (Crocetti et al., 2008; Meeus et al., 2014). However, some authors, like Erikson, have prominently featured the occupational identity (Kroger & Marcia, 2011). Theoretically, a well-established occupational identity allows the individual to make easy, rational, and mature career decisions (Skorikov & Vondracek, 1998; Vautero et al., 2018).

Work identity, occupational identity, vocational identity, and career identity are used interchangeably (Kielhofner, 2008; Skorikov & Vondracek, 2011). Work identity is associated with a job, occupational identity is generally associated with the identity of a specific profession (e.g., Skorikov & Vondracek, 2011) and vocational identity can be associated with students’ vocational decisions development (e.g., Green, 2020; Ouyang et al., 2021). In this study, we decided to use occupational identity, in coherence with the nomenclature adopted by Erikson (1968). It refers to self-awareness as a worker, representing the perception of occupational interests, capabilities, goals, and values (Kielhofner, 2008). Most authors on the subject consider it a central identity domain for many people, with a strong and extensive impact on people’s self-definition and well-being (Bowling et al., 2010). It is often conceptualized as the main component of general identity (Kroger, 2007; Skorikov & Vondracek, 2007). Erikson (1968) considers it the representation of a nucleus, an integrating element of identity, serving not only as a determinant of choice and performance of work, but also as an important factor in the emergence of meaning and structure in life. Thus, it is not surprising that the concept of occupational identity has been incorporated into almost every major career development theory (Skorikov & Vondracek, 2007).

In an attempt to conceptualize occupational identity, returning to Erikson (1968), the general identity emerges from the synthesis of a series of selves, abandoned and foreseen (Erikson, 1968), which will be integrated into a set of social roles. The totality of possibilities is the expression of diverse identities that people have through their lives, maturing and changing. What characterizes the definition of occupational identity is a clear sense of this trajectory and the possibilities that do not close. In this way, an adult occupational identity means: (i) a mature sense of occupational decisions, an understanding of who one is (commitment); (ii) a sense of desired and possible directions, an understanding of who one wants to be (exploration).

The operationalization of the Ericsonian occupational identity concept was most often guided by Marcia’s statutes (Marcia, 1966; Marcia, 1993). Marcia proposed two dimensions, exploration, and commitment. Exploration refers to the questioning and weighing up of various identity alternatives. Commitment refers to the choices made in relevant areas (Vautero et al., 2018). The association between exploration and commitment led to four identity statuses: achievement (high exploration and high commitment), moratory (low exploration and high commitment), foreclosure (high exploration and low commitment), and diffusion (low exploration and low commitment) (Marcia, 1966, 1993). This paradigm is still considered a valuable way to evaluate the occupational identity development and has guided several relatively recent works (e.g., Crocetti et al., 2008; Luyckx et al., 2006; Luyckx et al., 2005; Meeus et al., 2014; Vautero et al., 2018). The achievement state, characterized by high levels of career exploration and commitment, is a stable and mature identity status, though transitory (Germeijs & Verschueren, 2009).

Identity studies approach within career development field was always object of interest since the founder studies of the 1950 and 1960 decades, nevertheless, is possible to observe a growing interest in the last 5 years. Interestingly, most of those studies are associated with specific careers and are objects of interest of specialized journals, not in the career development field (Cake et al., 2020; Kirchknopf & Kögler, 2020). However, it is possible to identify some interest for study societies of developing economies that emphasize collective values (where the occupational identity development processes can involve diverse process or with different weights to each factor than in individualistic cultures (Ouyang et al., 2021). Occupational identity, in part is a response of the individual to a certain social context (Vondracek & Porfeli, 2011).

Despite internal differences, Brazil is a country with a population with a collectivist culture orientation (Gouveia & Clemente, 2000). (Byars-Winston & Rogers, 2019). Thus, due to the complexity of occupational identity development, it is possible to propose that in such cultures this process can be slightly, or not, different than in individualist cultures. For example, identity can be influenced by self-efficacy in the domain (Byars-Winston & Rogers, 2019). The latter is influenced by learning experiences, socially built, therefore, is possible to assume that cultural variances will have influence on occupational identity development.

## Occupational identity and socio-cognitive constructs

A number of studies have supported the proposition that adolescents and young adults who reported a strong occupational identity also reported good levels of career decision-making skills, career decision-making self-efficacy, career choice readiness, and career decidedness (e.g., Germeijs & Verschueren, 2009; Nauta & Kahn, 2007; Turner & Lapan, 2002). Choi et al. (2012) proposed that self-efficacy plays a leading role in vocational identity, as their meta-analytical study found an average *r* of 0.48 between identity and self-efficacy. This relationship could be explained based on the fact that the four experiential sources of learning theorized to build self-efficacy beliefs also contribute to identity (Gushue et al., 2006). For example, success experiences in any given academic field or performance accomplishments may strengthen an individual’s self-identification in that field (Gushue et al., 2006). Koumoundourou et al. (2012) reported that career decision-making self-efficacy mediated the association between core self-evaluations and occupational identity. Steiner et al. (2019) also confirmed this association.

However, few researchers focus on establish relationships between occupational identity and social cognitive career-related components, like self-efficacy and satisfaction in the career domain. An interesting study by Byars-Winston and Rogers (2019) posited that identity was influenced by self-efficacy in the domain, as an intermediary variable between individual’s learning experiences and people’s career intentions. This study also pointed out the potential contribution of some sources of learning, with special attention to cultural variances (Byars-Winston & Rogers, 2019).

On the other hand, occupational self-efficacy beliefs exert a strong influence on career, interests, goals, progress, and satisfaction (Lent et al., 1994; Lent et al., 2005), because establishing clear vocational interests and goals are core elements of a clear occupational identity. Occupational or academic self-efficacy beliefs are likely to be also highly relevant for the emergence of a clear occupational identity (Steiner et al., 2019). Consequently, those students with a positive sense of their competencies to deal with their educational tasks and barriers, for instance, would easily establish an occupational identity sense (Marcia, 1966; Steiner et al., 2019). People with high self-efficacy beliefs are more likely to be engaged in career exploration and commitment (Lee et al., 2018), which leads to a clear sense of occupational identity (Marcia, 1966).

Close to self-efficacy, some studies linked occupational identity to the perception towards academic or professional goals (e.g., Steiner et al., 2019), since clear goals are derived from a well-established occupational identity (e.g., Guan et al., 2015; Steiner et al., 2019). Students are more likely to be successful with their academic goals when they have a well-established occupational identity (Marcia, 1966). Occupational identity is an outcome of career development (Hirschi, 2013). Previously, Harren (1979) suggested that when a career decision is satisfactorily implemented, it leads to a stable occupational identity. Consequently, students who have a more positive notion of their competencies to deal with work and career-related challenges should more easily commit to an occupational identity (Steiner et al., 2019).

At the same time, identity can be associated with academic goals, and it is a well-established finding that identity statuses (Marcia, 1966) are distinguished by differences in well-being (Hirschi, 2011; Porfeli et al., 2011; Steiner et al., 2019). A clear occupational identity promotes adjustment and well-being in adolescents (e.g., Diemer & Blustein, 2007; Steiner et al., 2019). Probably such a relationship is established based on the idea that work and academic satisfaction are a key manifestation of domain-specific well-being (Lent & Brown, 2008). Lent et al. (2005) considered it plausible that satisfaction with a particularly valued life domain, like work or education, would relate more strongly to the influence of global life satisfaction. This assumption has been observed in a number of cross-cultural studies (e.g., Lent et al., 2012; Lent et al., 2018). Hence, the degree to which one likes or is happy with one’s school or work environment (Lent, 2013) can be influenced by occupational identity (Santisi et al., 2018). It is not possible to establish a causality, though an association has been observed in previous studies (e.g., Byars-Winston & Rogers, 2019; Steiner et al., 2019; Gushue et al., 2006; Lee et al., 2018).

Contextual variables play a significative role in the variables associated with occupational identity (e.g., self-efficacy, perceived progress in the domain, and satisfaction); that set of factors, like environmental supports and barriers, were directly associated with domain self-efficacy and goal progress, and indirectly associated with life and domain satisfaction by aiding or hindering goal pursuit and progress (Lent & Brown, 2008). Career barriers refer to negative contextual influences, such as events or conditions in the environment that make career progress difficult, as for example in unfavorable labor market conditions (Lent et al., 2000). Perceived barriers to career development can hamper career development, because people are unlikely to expend considerable resources to pursue their career interests and goal striving if they perceive barriers to their attainment (Lent et al., 2000; Steiner et al., 2019).

The perception of barriers was negatively associated with occupational identity differentiation among Latino high school students (Gushue et al., 2006). Steiner et al. (2019) found that perceived career barriers also hinder the emergence of a clear occupational identity. Students can expend less time and energy needed for the development of a clear occupational identity if they perceive barriers during their career’s development (Steiner et al., 2019). Consequently, if barriers can hinder career development process, environmental supports to cope with barriers can play a positive role in identity supports (Berríos-Allison, 2005; Luyckx et al., 2006) and cognitive variables, like self-efficacy (Lent, 2013; Lent et al., 2000).

## Purpose of the present study

**Fig. 1:**
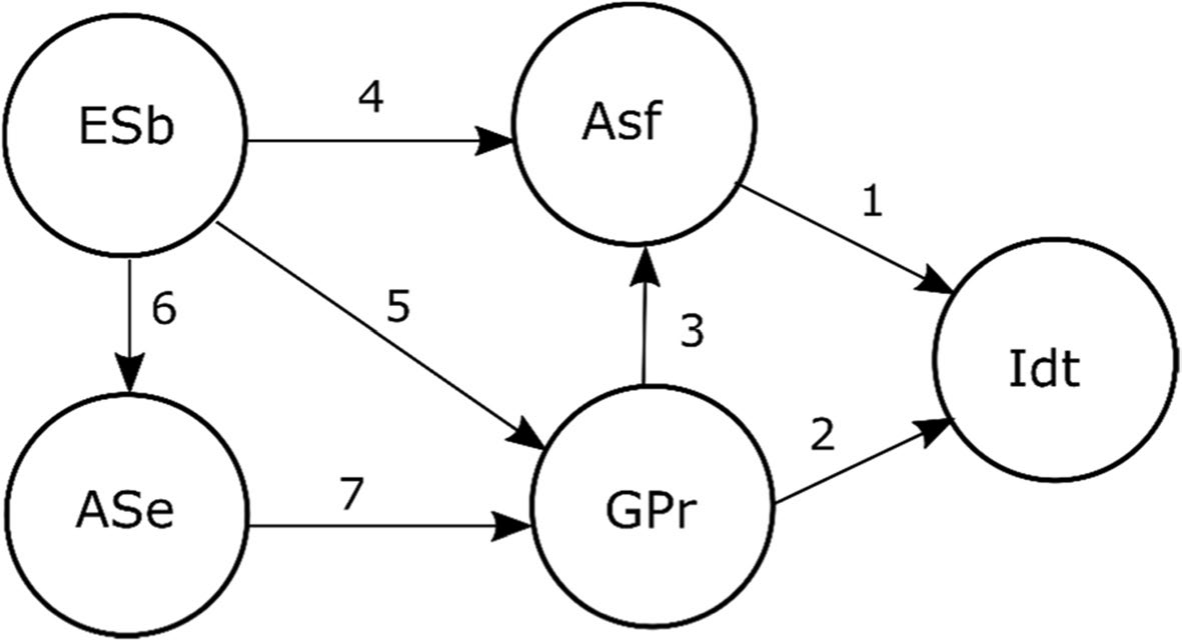
Proposed model and paths. *Note.* ESb, environmental support and barriers; ASe, academic self-efficacy; ASf, academic satisfaction; GPr, goal progress; Idt, occupational identity

Starting from the previous findings, this study assumes occupational identity as an indicator of career development and can be analysed as a result of the interactions among a set of socio-cognitive constructs that can be represented in a model. This, depicted in Fig. 1, postulates that a stable occupational identity is related to satisfaction in the academic domain (Path 1, Byars-Winston & Rogers, 2019; Steiner et al., 2019; Gushue et al., 2006; Lee et al., 2018) and to the perception of goal progress (Path 2, Hirschi, 2013; Guan et al., 2015; Steiner et al., 2019).

Academic satisfaction is also affected by goal progress (Path 3, Lent et al., 1994) and by the environmental supports and barriers (Path 4, Berríos-Allison, 2005; Luyckx et al., 2006; Steiner et al., 2019), which in turn also affect self-efficacy in the academic domain (Path 6, Choi et al., 2012; Germeijs & Verschueren, 2009; Nauta & Kahn, 2007; Turner & Lapan, 2002), as well as goal progress (Path 5, Lent et al., 1994). Additionally, the individual beliefs to progress in the academic domain will influence the perception about that progress (Path 7).

## Method

### Participants

The participants were 358 college students at a South Brazilian university, made up a non-probabilistic, intentional sample of 261 (72.9%) women and 97 (27.1%). The age range was 18–41 years old (*M* = 23.86, SD = 4.57). As for their current majors, 23.5% majored in Humanities or Arts, 21.8% majored in Social Sciences, 26% in Medical and Health Sciences, 17.3% in Engineering and Technology, 8.9% majored in Natural Sciences, and 2.5% in Agricultural Sciences. With regard to the year of study, 19.3% were freshman, 3.1% were sophomores, 5.6% were juniors, and 65.3% were seniors. We recruited a non-probabilistic, intentional sample of

### Instruments

#### Occupational identity

The Brazilian version (Vautero et al., 2018) of *Dellas Identity Status Inventory-Occupation* (DISI-0) (Dellas & Jernigan, 1981) was used to evaluate occupational identity. The scale is based on the Identity theoretical concept of Erikson (1968), as well as the identity status paradigm of Marcia (1966). The scale intends to identify the occupational identity of individuals in four statuses designed with Marcia’s original names and meanings. In this study, we focus on the Achievement status. The subscale of Achievement status is characterized by a high degree of exploitation of alternatives and commitment, and action on issues of occupational identity (Vautero et al., 2018). It is a 6-item, 5-Likert subscale (e.g., “I informed myself about the different career possibilities and now I can see myself working in what I have chosen”). This scale obtained Cronbach’s alpha 0.89 in the Brazilian version (Vautero et al., 2018).

#### Environmental support and barriers

Perceived environmental support and barriers in the academic domain were assessed with a Brazilian version (Lent et al., 2018) of a 9-item measure, listing a variety of conditions that may support students' academic career (e.g., “I am encouraged by my friends to go on with my studies”). Responses were obtained on a 5-point scale (1 = strongly disagree, 5 = strongly agree). Lent et al. (2005) found alpha values of 0.81 and reported that the scale was correlated with measures of self-efficacy, goal progress, and academic satisfaction. The Brazilian sample yielded an alpha value of 0.74 (Lent et al., 2018).

#### Academic self-efficacy

Self-efficacy was assessed with a Brazilian version of the academic self-efficacy measure used by Lent et al. (2005). The scale contains 12 items, divided into two subscales, designed to assess the degree of confidence that the individuals have in their ability to handle academic tasks (5 items, e.g., “deal with lack of support from the teachers or supervisors”) and to cope with barriers (7 items, e.g., “do well on your exams”). Responses were obtained on a 9-point scale (0 = no confidence, 9 = complete confidence). Prior versions of this measure have produced adequate internal consistency reliability estimates and shown a theory-consistent relationship with measures of academic outcomes (e.g., Lent et al., 2005). A study with a Brazilian sample yielded internal consistency estimates of 0.90 for the two combined scales (Lent et al., 2018). The scales about confidence in the ability to handle academic tasks yielded a coefficient alpha value of 0.89, and the scale about confidence to cope with barriers yielded 0.86 (Vautero et al., 2020).

#### Goal progress

The perception of progress in the academic domain was accessed by an instrument with 8 items developed by Lent et al. (2005), to assess how well students felt they were working towards different academic goals (e.g., “Completing all course assignments effectively”). Responses were obtained on a 5-point scale (1 = strongly disagree, 5 = strongly agree). The measure produced alpha values of 0.84 to 0.86 in two reported studies, and was correlated with measures of academic self-efficacy and satisfaction, outcome expectations, and environmental support (Lent et al., 2005). The Brazilian sample yielded a coefficient alpha value of 0.88 (Lent et al., 2018).

#### Academic satisfaction

Academic domain satisfaction was assessed with a 7-item measure using a 5-point (1 = strongly disagree, 5 = strongly agree) Likert-type scale (Lent et al., 2005). The measure asks participants to indicate the degree to which they felt satisfied with various aspects of their academic lives (e.g., “In general, I am satisfied with my academic life”). Lent et al. (2005) reported reliability estimates of 0.87 and correlation with measures of overall life satisfaction. The Brazilian sample yielded an alpha value of 0.83 (Lent et al., 2018).

### Procedure

The research plan obtained a formal approval from Research Ethics Committee of Federal University of Santa Maria (approval n° 17834819.5.0000.5346) that ensure all ethical research procedures with human participants.

The participants were recruited from college students at a South Brazilian university, all aged 18 years and older. Data collection was initiated after receiving institutional authorization, and the participants were recruited through email listservs of university students’ support services. A request for participation was sent using Surveymonkey. This is an online survey tool that was configured with informed consent, questions to collect demographic information (sex, age, school year, major, family education, and occupation), and the psychological instruments. The data was collected at the end of the spring semester and the beginning of the autumn semester of the 2016/2017 school year. Participation was voluntary and anonymous. Some surveys had missing data, and following best practices to deal with incomplete surveys, those with more than 10% of missing answers were removed (Schlomer et al., 2010). Were received initially 372 surveys, 14 of them with missing data per case above 10%. After removing these cases, in the remaining 358 surveys, 4 variables showed items with missing data (portion < 0.06) in the remaining questions, and, for this reason, multiple imputations for dealing with this missing data were used (Little & Rubin, 2002).

The proposed model performed a path analysis, using the covariance matrices of the observed variables and maximum likelihood estimation; the analyses were computed using the Statistical Package for Social Sciences version 20 (IBM SPSS Statistics 20, SPSS Inc., Chicago, IL) and the AMOS statistical package version 21.0.

Complete models within SEM are composed of two models, a measurement model and a structural model (Byrne, 2010). The measurement model defines the relationships between observed and unobserved variables. Different models’ fits were tested and compared in terms of chi-square/degrees of freedom ratio (*χ*^2^/df), the comparative fit index (CFI), the root mean squared error of approximation (RMSEA), and the standardized root mean squared residual (RMR). According to Hu and Bentler (1999), good model-data fit was inferred from RMR values close to 0.08, in combination with CFI values close to 0.95 or RMSEA values close to 0.06. The *χ*^2^/df values smaller than 2 were considered as indicators of a good fit (Byrne, 2010), values between 2 and 5 as acceptable, and larger than 5 as unacceptable (Marôco, 2010).

## Results

Before starting the analysis of the measurement and structural model, some sociodemographic characteristics are presented. The parental education is relatively similar for fathers and mothers, though mothers’ education level was slightly superior, as presented in Table 1. Half students came from families with less than four people, the other half had between four and six persons (Table 2). Most families were considered conjugal families (57%, *n* = 205), followed by single parent families headed by women (25.4%, *n* = 91), other configurations represented only 6.1% of the cases (*n* = 22). Information was not available 11.2% of the cases (*n* = 40).

**Table 1:** Parents education

Educational level	Father		Mother	
	*n*	*%*	*n*	*%*
No formal education	2	0.56%	5	1.40%
Primary	102	28.49%	140	39.11%
Secondary	115	32.12%	109	30.45%
Tertiary	61	17.04%	81	22.63%
Does not apply	78	21.79%	23	6.42%

**Table 2:** Students’ sociodemographic characteristics

Baseline characteristic	*n*	*%*
Family size		
1–3 persons	159	44.41%
4–6 persons	158	44.13%
More than 6 people	2	0.56%
Without information	39	10.89%
Main source of income		
Own resources	106	29.61%
Family support	226	63.13%
Social aids	25	6.98%
Without information	1	0.28%
Local of residence		
Rural communities	59	16.40%
Small/medium towns	122	34.10%
Large city/large town	164	45.80%
Without information	13	3.70%

Family support was the main source of income for students, however, students who lived by their own resources were almost one third of the sample, as can be seen in Table 2. The place of origin of most students were relatively large towns, with more than 50,000 un-inhabitants.

The assumptions for Structural Equation Modelling were previously analysed, namely, the linearity of the relationships and the normality of the variables (Byrne, 2010). The normality of the variables was estimated, first, by the results of Mardia’s multivariate kurtosis, which did not obtain values greater than three, indicating no normality problems (Ullman, 2006). However, since this coefficient may not be reliable in samples with more than 200 cases (Kline, 2016), the univariate and multivariate asymmetry (Sk) and kurtosis (Ku) coefficients were observed. Asymmetry measures (sk) between the variables were not greater than 3, and kurtosis (ku) did not show values above 7; therefore, removals were not necessary (Marôco, 2010).

First, a measurement model that represented the five theoretical constructs (occupational identity, self-efficacy, goal progress, environmental support, and academic satisfaction) was tested as separate, but correlated latent dimensions.

The instruments had a high number of items; thus, it was decided to group the items of four instruments into parcels, in order to reduce common problems of variance in these cases (Little et al., 2002). These parcels were built based on an exploratory factor analysis of the items in each scale and considering the dimensionality of the scales (Little et al., 2002). Parcels were formed according to the magnitude of the factor loading, in order to have relatively equal loadings for each parcel (Little et al., 2002). Self-efficacy was represented by two indicators: academic milestone and coping self-efficacy scale scores. Occupational identity was represented by two indicators with 3 items per parcel. Academic satisfaction, environmental supports and barriers, and goal progress were each represented by two indicators, with 2–3 items per parcel. After the parcels were created, a Confirmatory factor analysis using maximum likelihood estimation was conducted, with items/parcels assigned to the appropriate latent construct. The seven-factor measurement model produced good fit to the data in the full sample: CFI = 0.97, GFI = 0.93, RMSEA = 0.050, SRMR = 0.036, *χ*^2^/df = 1.82. Coefficient alpha means, standard deviations, and correlations among variables are shown in Table 3.

**Table 3:** Means, standard deviations, and correlations among variables

	Idt	ASe	ASf	ESB	GPr	SAT	SCB	*M*	SD	α
Idt	–	–	–	–	–	–	–	3.45	.90	.89
ASe	.40**	–	–	–	–	–	–	7.06	1.56	.90
ASf	.48**	.55**	–	–	–	–	–	3.80	.81	.83
ESB	.34**	.46**	.65**	–	–	–	–	3.75	.72	.74
GPr	.39**	.65**	.65**	.54**	–	–	–	3.77	.79	.88
SAT	.48**	.88**	.56**	.42**	.68**	–	–	7.40	1.69	.89
SCB	.31**	.94**	.46**	.41**	.53**	− .20**	.41**	7.82	1.70	.86

*Note.* **p < .01; *p < .05. *Idt* identity, *ASe* academic self-efficacy (composite), *ASf* academic satisfaction, *ESB* environmental support and barriers, *GPr* goal progress, *SAT* self-efficacy in academic tasks, *SCB* self-efficacy to cope with barriers

## Test of the structural model

The hypothesized model (M1) had a poor fit, *χ*^2^/df = 5.19, CFI = 0.98, RMR = 0.05, and RMSEA = 0.11. It is recommended that alternative models be tested, especially given the complexity of the hypothesized model (MacCallum et al., 1996). For this purpose, the self-efficacy function and representation was analysed. This variable was hypothesized as a central component in the proposed model (Steiner et al., 2019), related to how confident students were in their abilities to perform a variety of behaviours required for general academic success and progression (Lent et al., 2005). The scale used in M1 considered a measures of two subscales of self-efficacy, self-efficacy for completing broad academic milestones and academic coping efficacy. This procedure was adopted with previous studies (Lent et al., 2005). With the purpose of better explore the sub-scale’s function, two alternative models were built, firs using only measures of self-efficacy related to academic milestones (M2) and then, a model using measures of academic self-efficacy to coping with barriers (M3). Model 3 had a better fit than the other possibilities, as shown in Table 4, with improvement in all indices and a far greater difference in the Akaike index (AIC = 30.324).

**Table 4:** Summary of fit indices for models’ comparisons (*n* = 323)

Model	(χ^2^/d.f.)	*χ*^2^	CFI	RMSEA	SRMR	AIC
				(90% CI)		
Model 1: academic self-efficacy (composite)	5.19	15.5	.98	.11	.05	39.58
Model 2: Self-efficacy in academic tasks	8.32	77.3	.97	.15	.07	48.957
Model 3: Self-efficacy to cope with barriers.	2.11	6.33	.99	.06	.04	30.334

*Note*. *CFI* Comparative Fit Index, *RMSEA* the root mean square error of approximation, *SRMR* the standardized root mean square residual, *AIC* Akaike Information Criterion

**Fig. 2:**
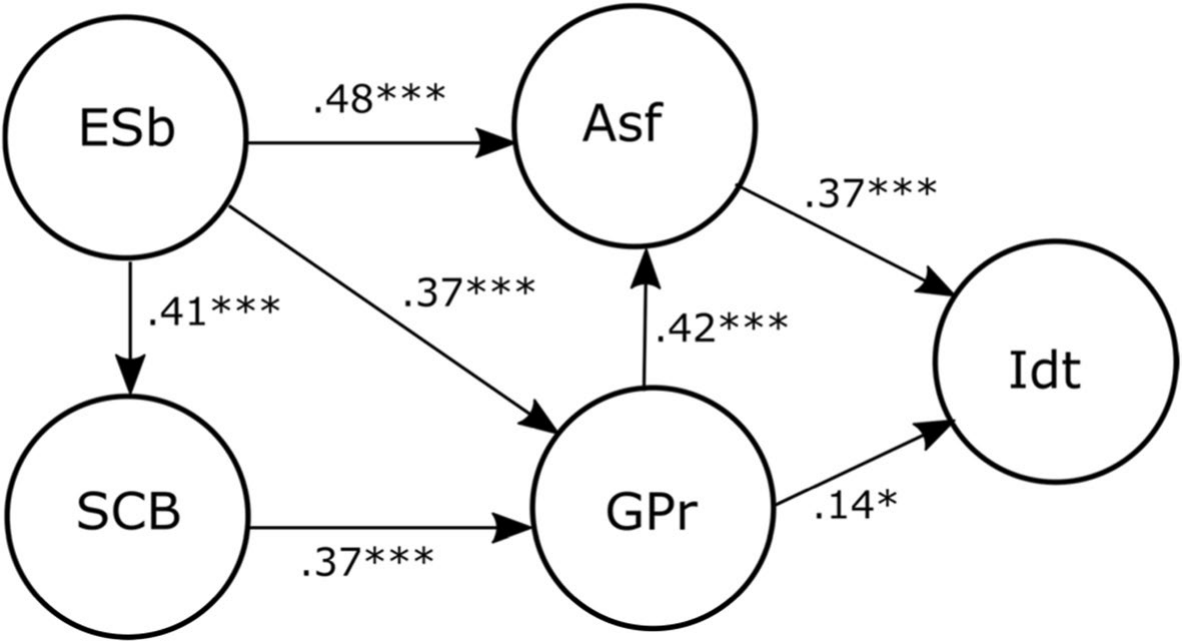
Factorial weights of model variables. *Note.* ESb, environmental support and barriers; SCB, self-efficacy to copy with barriers; ASf, academic satisfaction; GPr, goal progress; Idt, occupational identity. *p < .05, ***p < .001

All hypothesized paths yielded statistically significant coefficients, as shown in Fig. 2. The occupational identity was partially well predicted by the combination of self-efficacy to cope with barriers, support, academic satisfaction, and goal progress (*R*^2^ = 0.23). The structural model also accounted for substantial portions of the variance in academic satisfaction, goal progress and self-efficacy to cope with barriers; *R*^2^ values were, respectively, 0.55, 0.41, and 0.17.

Factorial weights values indicate that changes in academic satisfaction are associated with higher changes in identity than a change in goal progress, and those results can indicate a strong direct role of satisfaction. However, the indirect effect still suggests a relevant factor to analyse whether occupational identity was indirectly associated with the perceived support and barriers in the academic domain, and the beliefs to cope with tasks and barriers in the academic domain (self-efficacy), through the perceived progress and satisfaction in the same domain.

## Discussion

This study aimed to demonstrate how occupational identity can be seen as a result of career development through a set of socio-cognitive factors interaction. To fulfil this purpose, a model was proposed with direct associations from occupational identity to the perceived progress and satisfaction in the academic domain, and indirect association to perceived support and barriers, and the beliefs to cope with tasks and barriers in the same domain.

In order to evaluate such associations, a model was proposed with paths that describe how the occupational identity of college students is associated with indicators of satisfaction and progress in the academic domain, contextual support and barriers, and self-efficacy beliefs. Concretely, based in Lent (Lent, 2013; Lent et al., 1994; Lent et al., 2005), it was proposed that the students’ occupational identities are defined and well established, to the extent that they are happy in the academic domain and are involved in activities they value, see themselves as making progress at personally relevant goals, possess strong self-efficacy at performing necessary tasks and at achieving their goals, and have access to resources in the environment for promoting their self-efficacy and aiding their goal pursuit.

For this purpose, the study tested a model, and the results highlighted that the self-efficacy to coping with barriers had a better fit than the other possibilities (Model 1: Academic Self-efficacy composite and Model 2: Self-efficacy in Academic Tasks). These results highlight the importance of confidence in dealing with barriers in higher education. Thus, the level of challenge provided to students should be considered, in order to allow them space for growth, as well as avoid successive frustrating events that can affect their beliefs of self-efficacy.

These results are supported by literature that indicates that students with a positive sense of their competencies to deal with their educational barriers, for instance, would easily establish an occupational identity sense (Marcia, 1966; Steiner et al., 2019). People with high self-efficacy beliefs are more likely to be engaged in career exploration and commitment (Lee et al., 2018), which leads to a clear sense of occupational identity (Marcia, 1966).

There are some points to highlight in the final model, for example, the role of perceived barriers, which was negatively associated with occupational identity differentiation in previous studies (e.g., Gushue et al., 2006). Steiner et al. (2019) proposed that students might expend less time in developing clear occupational identity if they perceive barriers. In the present study, it was noted that even in the presence of perceived barriers, with adequate support and a positive sense of competence to deal with such barriers, those barriers did not hamper the occupational identity development.

Another relevant issue is the role of academic satisfaction, as previous studies indicated the association between a clear occupational identity and well-being (e.g., Diemer & Blustein, 2007; Steiner et al., 2019). The well-being in a specific domain, like work or education, would relate more strongly with occupational identity than general well-being (e.g., Lent et al., 2012; Lent et al., 2018). Indeed, some studies posited that occupational identity promotes well-being (e.g., Steiner et al., 2019).

## Conclusions

This study proposed a reverse causality and tested occupational identity as an output of career development factors, among them, academic satisfaction (Harren, 1979). In fact, previous studies proposed a strong association, but without indicating causality (e.g., Byars-Winston & Rogers, 2019; Gushue et al., 2006; Lee et al., 2018; Steiner et al., 2019).

These results reinforce the relationship between career development and academic development in higher education as suggested previously (Gushue et al., 2006), providing clear clues for policies and career interventions in this context of education. For example, the present findings point to the indirect influence of supports to deal with barriers, and self-efficacy to cope with barriers in the occupational identity development. Prior works have indicated that students with a positive sense of their competencies to deal with academic barriers are more likely to easily establish an occupational identity sense (Marcia, 1966; Steiner et al., 2019). In this sense, strategies to cope with barriers of academic life can be a target of intervention and a way to develop a sense of positive occupational identity in college students. Providing support and helping students handle difficulties in academic life can be the focus of student service interventions, mostly aimed at freshman and sophomores.

In the world of work, vocational identity has influences on several implications. Occupational identity influences the success of occupational adaptation, that is, it influences the achieving of occupational competence over time (Kielhofner, 2008). In that way, the occupational identity will have an impact on junior and senior students, and probably post-graduates, in a different way than on younger ones. Probably, the barriers students have foreseen and experienced will be related to future jobs and professional career, and interventions with these students can be aimed to help them transition from college to the workplace. This transition will not create a new occupational identity, but new opportunities for explorations and new commitments (Luyckx et al., 2005). However, the elements pointed out here, mainly the role of the barrier, the ability to cope with such barriers, and the satisfaction with the progress in the domain, are still key targets of career interventions.

## Limitation and future research

This study has some limitations, but they are understood as challenges for further research. First, all the obtained data were collected in just one time point. Vocational identity has a complex process development over time, at different points of academic life, so students are likely to have different levels of identity development. Future research would benefit from multiple time-points assessments. However, to determine the exact development of occupational identity over time, it would be relevant to observe what is happening in the environment surrounding the student. Lack of support from professors and new roommates are examples of possible barriers to occupational identity development. Another limitation, but at the same time an opportunity for future research, is the association between academic satisfaction and identity. Past research posited an acyclic association (Steiner et al., 2019), from identity to satisfaction, and this study tested the contrary association. Nevertheless, it is possible to propose other possibilities; for example, a feedback loop between those variables is a possibility.

The validation study for DISIO-O was carried out within the same sample of this work. It is a recommendation in the literature to split the sample, or use two sample, to conduct these studies. However, considering that in the mode evaluated the interest was to evaluate the adjustment indices for comparative purposes, the strategy may not represent a problem (Fabrigar & Wegener, 2014). Nevertheless, it is a limitation and the results cannot be generalized.

### Practical implications

Despite the limitations identified in the current study, it brings some different possibilities of understanding into an area with growing interest. Identity as a developmental variable depends on, in part, of environmental/contextual influences. In this study, the contextual variables were represented by the environmental supports and barriers. These influences were significative for Brazilian students; however, the occupational identity development in other cultures can follow different patterns or have different weights for each variable. In other words, the identity formation within the career development should be interpreted in the light of the culture it grows. Consequently, career interventions aiming identity development should be tailored for each culture, or better, developed within the context of interest.

## Data Availability

The data that support the findings of this study are available from the corresponding author, JRV, upon request.
